# Notes and News

**Published:** 1903-02-21

**Authors:** 


### NOTES AND NEWS.

HOSPITAL MEETINGS AND FESTIVALS.

Queen Charlotte's Hospital.—Annual Meeting, Monday, February 23rd, at 3 P.M.

Tottenham Hospital.—Annual Meeting, Tuesday, February 24th, at 5.15 P.M.

Cancer Hospital.—Annual Meeting, Wednesday, February 25th, at 4 P.M.

Middlesex Hospital.—Annual Meeting, Thursday, February 26th at 12 noon.

East London Hospital for Children.—Annual Meeting, Thursday, February 26th, at 3.30 P.M.

King's College Hospital. — Annual Meeting, Thursday, February 26th, at 4 P.M.

Royal Orthopædic Hospital.—Annual Meeting, Thursday, February 26th, at 4.30 P.M.

Seamen's Hospital.—Annual Meeting, Friday, February 27th, at 3 P.M., at the Institute of Chartered Accountants, Moorgate Place.

Great Northern Central Hospital. — Annual Meeting, February 27th, at 5 P.M.

A NOVEL use of a limestone cave has been made in Virginia. The air in these caves is found to be remarkably pure and healthy, and at Luray the cave has been utilised to supply a sanatorium with air, each room getting its supply direct.

DRUG adulteration is carried on to a large extent in America. A recent analysis showed that out of 373 samples of phenacetin purchased at different chemists only 58 were pure, whilst many of the preparations contained no phenacetin whatever.

SIR JOHN LENG, M.P., will preside at the sixty-fourth anniversary festival in aid of the funds of the Newsvendors' Benevolent and Provident Institution, to be held at De Keyser's Royal Hotel, Victoria Embankment, London, E.C., Tuesday evening, May 12th.

NOW that the subject of the water supply of the Metropolis is again exciting the attention of the County Council, the announcement by The Scientific Press, Ltd., of a cheap edition (10s. 6d. net) of Dr. Sisley's book, dealing with this much vexed question, is a welcome one. Dr. Sisley is an acknowledged authority on this subject, having given years of study to it.

THE St. Petersburg Institute of Medical Women has been open for three years. Eleven of the students have received diplomas qualifying them to practise medicine.

LORD ROBERTS has joined the new Temperance League, which limits the use of alcohol to meal-time. There can be no doubt that the support of persons who can command public confidence and admiration will do much to encourage the weak and half-hearted.

MR. G. Q. ROBERTS, who has resigned the post of house governor and secretary of the London Hospital, has been appointed secretary of St. Thomas's Hospital. The post as now constituted is practically a new office, as with the control of the secretariat is now combined the supervision of the landed property of the institution.

AN Industrial Sanatorium Farm Colony has just been opened at Ipsden, near Wallingford, by Drs. Charles Reinhardt and Frank Fowler (who conduct the Hailey Sanatorium, near Wallingford, and the Stourfield Park Sanatorium, Bournemouth, for wealthier patients). At the subscription sanatorium patients are received at a small charge, and are expected to work at such employment as the resident physician may find them physically and otherwise fit for, and amongst other avocations they have the opportunity of learning pheasant-farming, poultry-rearing, gardening, and various agricultural operations, in order to gain experience in such outdoor occupations as may be useful to them after they leave the sanatorium. This industrial employment added to the sanatoria treatment is an excellent idea. The drawback in sanatoria treatment is that it is efficacious in many cases only while it lasts, and that the money expended only helps to delay the fatal ending for a short time where patients have to return to unhealthy occupations and dwellings. Many persons fall victims to their surroundings who would long continue useful members of the community if healthily situated.

The *Army and Navy Gazette* points out that "although among the *ex-officio* members of the Commissioners for the Royal Hospital Chelsea a military medical member—viz. the Director-General of the Army Medical Service—finds a place, yet among the *ex officio* members of the Commissioners of the Duke of York's Royal Military School no military medical member has been appointed." It goes on to say: "Surely one is quite as necessary in the latter institution as in the former. This will have to be remedied without delay, and we understand that a question on the subject will arise when Parliament assembles. If the Chaplain-General and Judge-Advocate-General take places as *ex-officio* members of the Duke of York's School, surely a military medical member of rank and experience should be appointed for the sanitation of the institution, and matters connected with the health of the boys must come up now and again for consideration. It is now well known that the sanitary condition of the Duke of York's School has been recently investigated, and it is not unlikely that the Secretary of State for War is, by this time, in possession of the report thereon, the particulars of which will be called for on the reassembly of Parliament."

